# Rare Case Report of an Endometrial Adenocarcinoma Arising in a Complete Septate Uterus With a Double Cervix and Vagina

**DOI:** 10.7759/cureus.10382

**Published:** 2020-09-11

**Authors:** Obed Rockson, Abdelbassir Ramdani, Tariq Bouhout, Badr Serji, Tijani El Harroudi

**Affiliations:** 1 Surgical Oncology, Mohammed VI University Hospital, Regional Oncology Center, Oujda, MAR

**Keywords:** endometrial cancer, congenital malformation, septate uterus, cervical duplication, vaginal duplication

## Abstract

Endometrial adenocarcinomas arising in a complete septate uterus with cervical and vaginal duplication are rare. Here, we report a case of stage III endometrioid endometrial adenocarcinoma arising in a complete septate uterus with a double cervix and vagina coupled with a left serous ovarian cystadenoma in a 35-year-old-female patient. The patient underwent a total abdominal hysterectomy with bilateral salpingo-oophorectomy and was addressed to the oncologist for adjuvant radio-chemotherapy. We highlight the symptoms, diagnosis, and therapeutic management, and compare them to the recent literature.

## Introduction

The uterus is the fourth most common primary site of cancer among women, and more than 80% of uterine cancers are endometrial adenocarcinomas [1]. Congenital malformations of the female reproductive tract are frequent, noticeable in 3-4% of the general population, and 7-9% of the infertile population [2, 3]. There are several kinds of classification published for female congenital genital anomalies, also called Müllerian anomalies, which are based on the extent of Müllerian duct development and fusion. Septate uterus is the most common congenital uterine duplication anomaly, representing approximately 55% of Müllerian ducts anomalies [4]. It is caused by the incomplete fusion of the Müllerian ducts resulting in two uterine cavities with a single fundus, cervix, and vagina, which can also be separated. The occurrence of endometrial adenocarcinoma on a complete or partial septate uterus, with or without cervical and vaginal duplication, is extremely rare. We report a case of an endometrial adenocarcinoma arising in a complete septate uterus with a double cervix and vagina coupled with a left serous ovarian cystadenoma in a 35-year-old-female patient.

## Case presentation

A 35-year-old female patient (gravidity 1; parity 0) with a body mass index (BMI) of 29.6kg/m^2^ was referred to our hospital for complaint of dysfunctional uterine bleeding for two months. She had her menarche at the age of 14, with regular cycles, slight dysmenorrhoea, and dyspareunia. Her past medical history was noticeable for a miscarriage after eight weeks of gestation and a five-year history of type 2 diabetes under insulin therapy. There was no family history of any congenital anomalies, and the patient was unaware of her uterine anomaly. Gynecologic examinations revealed a phenotypic female (Tanner stage V), a soft abdomen with no tenderness on palpation of the bilateral adnexa, a normal with double cervices, a double vagina with a longitudinal vaginal septum, no contact bleeding, and the external genitalia was normal. We had no previous information concerning her gynecologic examination in between her miscarriage and bleeding. 

**Figure 1:**
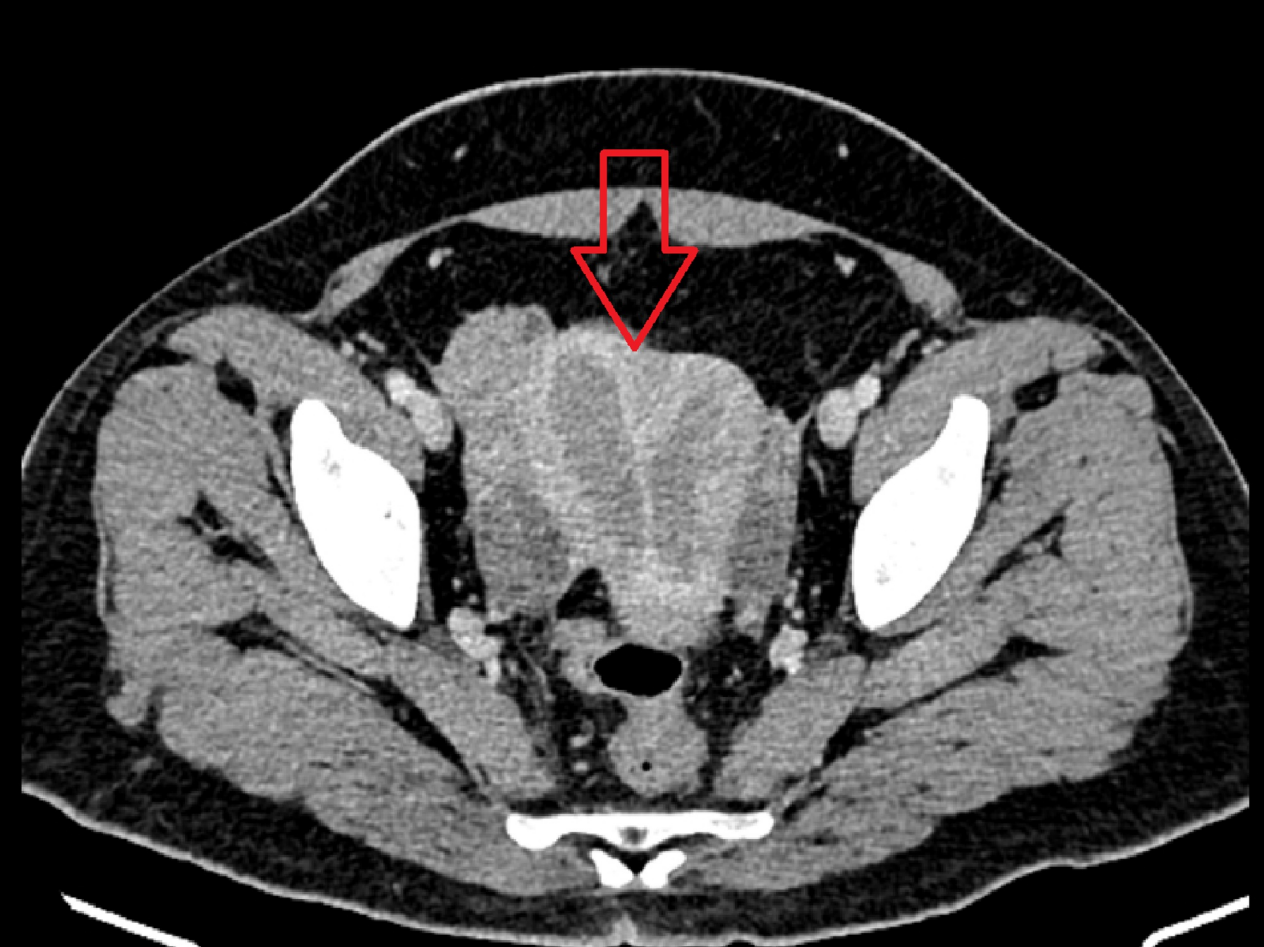
CT scan showing the presence of the tumor invading both uterine cavities with thickened walls (red arrow)

Hysteroscopy was performed with uterine endometrial biopsy, which revealed a malignant tumor, suggestive of moderately differentiated adenocarcinoma. Genetic counseling for cancer was unavailable, and ultrasound was not obtained. Abdomino-pelvic contrasted computerized tomography (CT) scan revealed a slightly increased size of the uterus, 87x37x70mm, double uterine cavities, raising the concern for stage III endometrial tumor thickening invading more than 50% of the thickness of the uterine muscle, with the onset of bilateral invasion of the parametria, and small lymph nodes without infiltrating the cervix (Figure 1).

**Figure 2:**
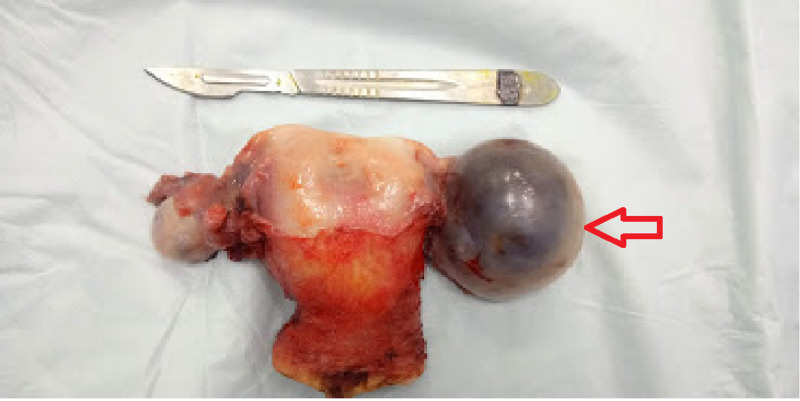
Surgical specimen of the removed septate uterus with grossly visible left cystic ovarian lesion (red arrow)

**Figure 3:**
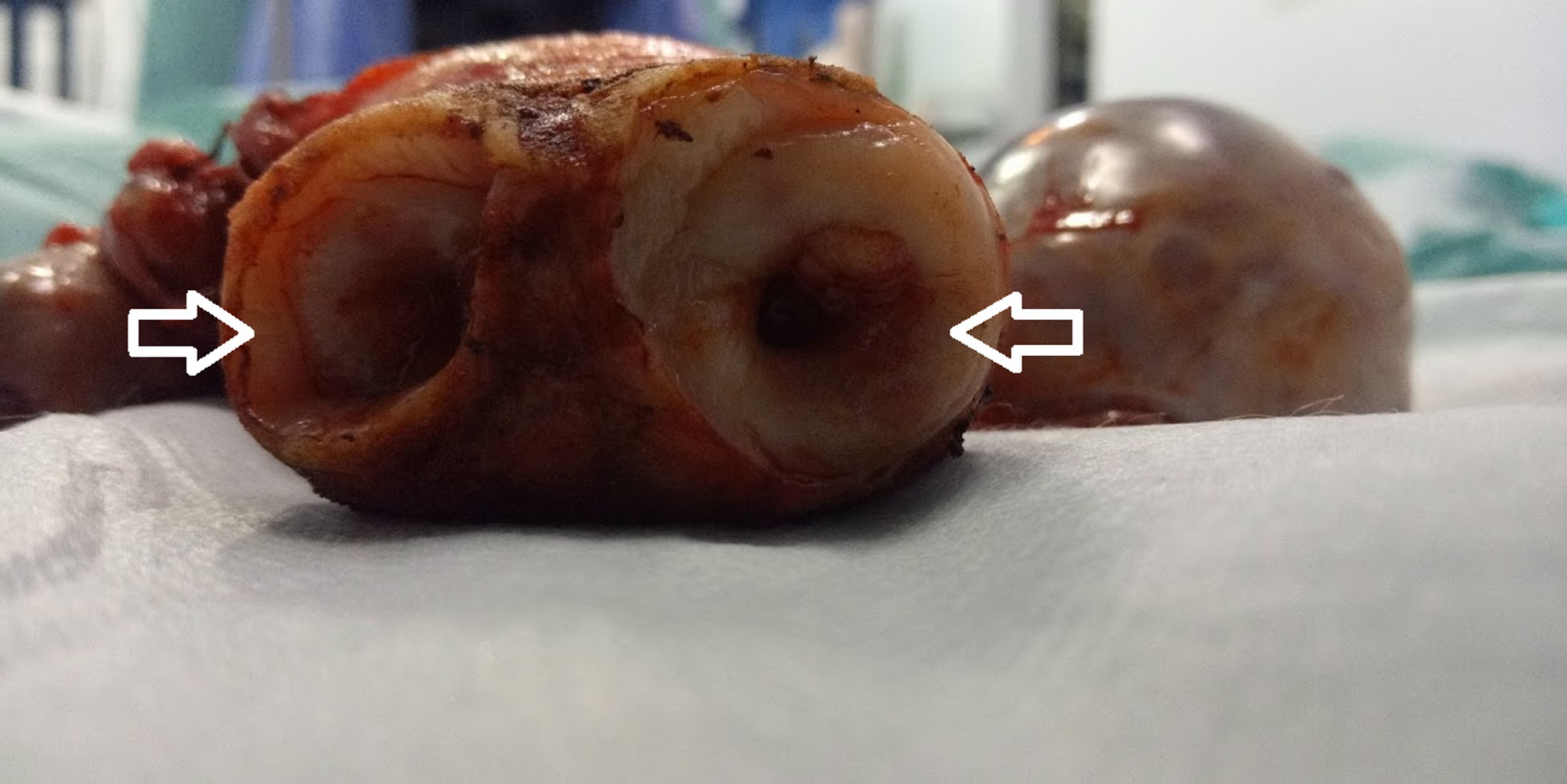
Surgical specimen showing the double cervices (two white arrows)

**Figure 4:**
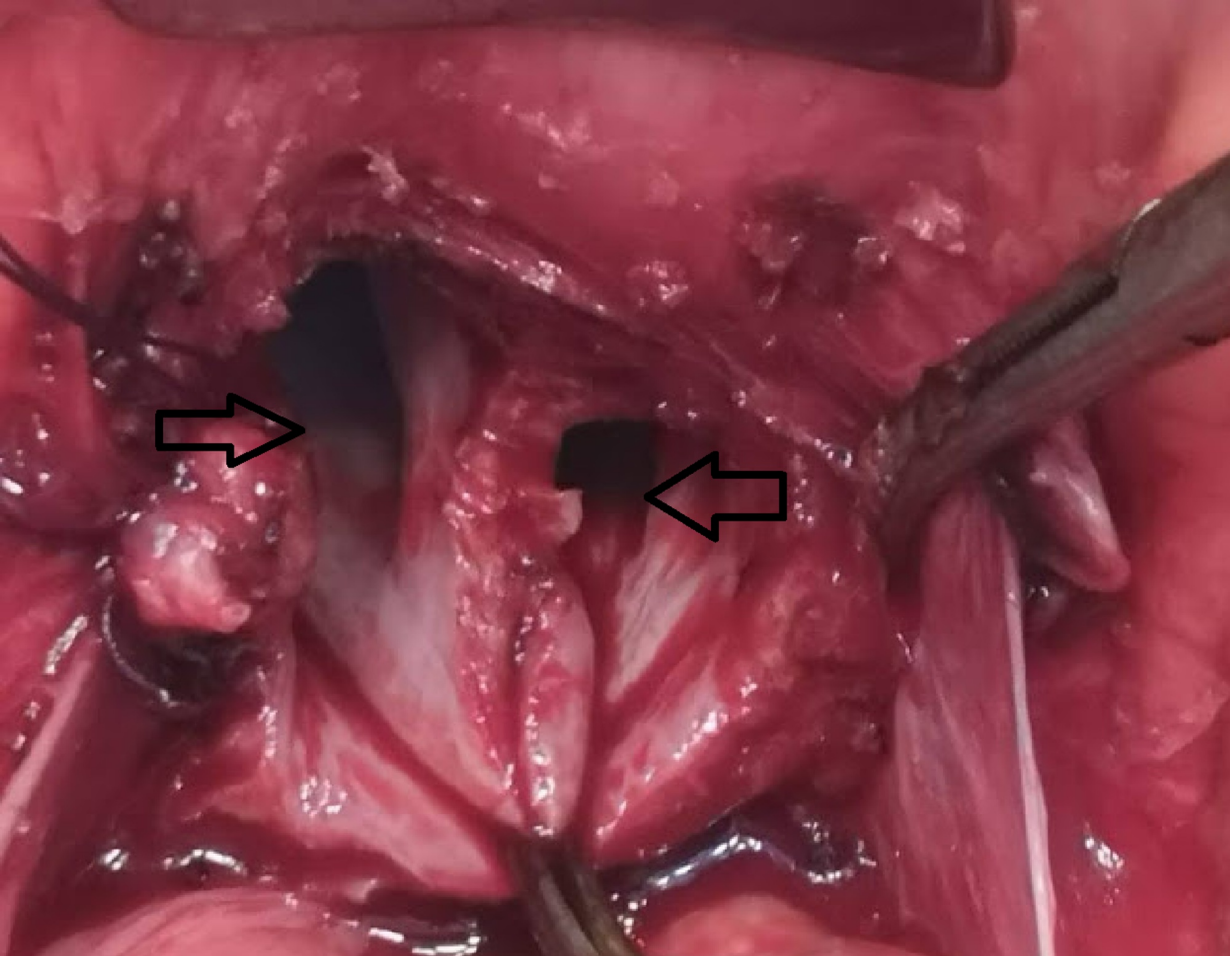
Intra-operative surgical view showing the vaginal duplication (two black arrows)

After a complete anesthesia fitness, glycemic control with insulin, and informed consent, the patient underwent an exploratory laparotomy where a total abdominal hysterectomy, bilateral salpingo-oophorectomy, and staging workup (bilateral pelvic and paraaortic lymph node dissection) were performed. At the time of surgery, the unusual per-operative finding was that of a septate uterus with complete duplication of the cervix and vagina (Figures 2-4). 

The patient’s postoperative course was uneventful. Gross examination revealed a complete septate uterus, with a complete septum from the fundus to cervix measuring 6x6x3.5cm and a double cervix measuring 4x2.5x3cm for the largest. Microscopic examination revealed, as designated by the International Federation of Gynecology and Obstetrics (FIGO), a grade II endometrial endometrioid adenocarcinoma, infiltrating the entire uterine wall coming into contact with the serosa, the isthmus, and the lymph nodes, with a left serous ovarian cystadenoma.

The disease was consistent with FIGO stage IIIC (pT1bN1Mx). She recovered rapidly from surgery and was addressed for adjuvant combined radio-chemotherapy to the pelvic and para-aortic region for high-risk endometrial cancer. She received a total dose of 46Gy of external beam radiotherapy in two fractions given on five days per week, two cycles of cisplatin 50mg/m^2^ given intravenously during radiotherapy, followed by four cycles of carboplatin and paclitaxel 175mg/m^2^ given intravenously. A follow-up period of eight months has shown no signs of recurrence.

## Discussion

To discuss this rare condition, we performed a literature review of cases of endometrial carcinoma (EC) associated with Müllerian duct anomalies (MDA). Müllerian anomalies have not been implicated as a significant risk factor for the development of cervical, uterine, and ovarian cancers; however, several reports describe the existence of various tumors in anomalous uteri [5-7]. We defined endometrial adenocarcinoma associated with MDA involving all the seven most common anomalies, as developed by the American Society of Reproductive Medicine [8]. We used multiple keywords in different combinations on the available databases and included articles in both English and French literature between January 1990 and May 2020.

A total of 27 cases of EC arising within MDA (including our case) have been reported. Of the 27 patients, the ratio of uterus didelphys was as high as 48.1% (13/27), bicornuate uterus was 33.3% (9/27), and septate uterus was 14.8% (4/27). EC arising in the septate uterus is rare, and the present report represents the fourth of such cases being reported in the literature. Itoh et al. described the first case in 2014 in a 39-year-old patient with a septate uterus who presented with postpartum bleeding. The histological diagnosis was consistent with well to moderately differentiated, endometrioid adenocarcinoma arising in both cavities of a septate uterus, which was detected six months after full-term delivery [9]. The second case was reported by Boubess et al. in 2015 in a 67-year-old patient with EC located in the right cavity of a complete septate uterus. The histopathological diagnosis found a grade I endometrioid adenocarcinoma, which infiltrated more than one half of the myometrium [10]. The third case was published by Gao et al. in 2017 which involved a nulliparous 60-year-old patient with a septate uterus, and the final histopathological findings confirmed a grade II endometrioid adenocarcinoma in the left cavity, invading less than half of the myometrium. No adjuvant therapy was administered, and disease-free survival was two years [11]. We summarized the diagnosis, outcome, symptoms, diagnosis of histopathological examination, surgical stage (base on FIGO, 2008), surgical treatment, and adjuvant therapy for these four cases (including this present case) in Table 1.

**Table 1:** Comparing reported cases of EC located in septate uterus for the past three decades to our case EC - endometrial carcinoma; MDA - Müllerian duct anomaly; EAC - endometrial adenocarcinoma; SU - septate uterus; FIGO - International Federation of Gynecology and Obstetrics; CT - chemotherapy; VS - vaginal septum; DV - double vagina; DCs - double services; OCA - ovarian cystadenoma; N/A - not available in the original article

No. Author (year)	Age	Gravidity/parity	Medical history	Symptom	Histology	MDA	FIGO	Surgery	Adjuvant therapy	Survival
1. Itoh et al. [9] (2014)	39	G2P2	Unremarkable	Bleeding	EAC in both cavities with focal squamous differentiation	SU	Stage Ic grade III	Staging surgery	CT	three years
2. Boubess et al. [10] (2015)	67	G0P0	Unremarkable	Bleeding	EAC of the right cavity	SU	Stage: N/A Grade I	Staging surgery	N/A	N/A
3. Gao et al. [11] (2017)	60	G0P0	Hypertension + obesity	Bleeding	EAC of the left cavity	SU + VS	Stage IA, Grade II-III	Staging surgery	none	two years
4. Present case	35	G1P0	Diabetes mellitus + overweight + miscarriage	Bleeding + pelvic pains	EAC in both cavities + left OCA	SU + DCs + DV	Stage IIIC Grade II	Staging surgery	CT + RT	eight months

The behavior and incidence of EC arising in a septate uterus have not been well documented and less in its association with ovarian cystadenoma. Women with MDA appear to have a higher rate of unexplained infertility, endometriosis, spontaneous abortion, breech presentation, and premature delivery [12, 13]. Those with a history of miscarriage (as was in our case) have been found to have a significantly increased incidence of uterine abnormalities. Major uterine malformations occur in only 0.5-5% of the general population, 0.1-3% of fertile women, 3% of infertile women, and 5-10% of women who have miscarriages [2, 3, 13]. The majority of cases of endometrial cancer are diagnosed in early stages, as abnormal uterine bleeding is the presenting symptom in 90% of cases [7-10]. Relevant to this present case, abnormal uterine bleeding with pelvic pains was the mode of revelation. Obesity/overweight, diabetes mellitus, infertility, and nulliparity should also be considered as risk factors for uterine neoplasm in this patient. 

Pelvic ultrasonography, CT scan or magnetic resonance imagery (MRI), hysteroscopy, and dilatation and curettage (D&C) or endometrial biopsy are all among the diagnostic evaluation of EC. Pelvic MRI is the investigation of choice for detection of MDA with an accuracy of up to 100% being reported [4]. It is useful in diagnosing malignant tumors of the uterus and provide valuable imaging modality for pre-surgical locoregional staging of EC. Our patient underwent a CT-scan examination and an endometrial biopsy in which the uterine anomaly and endometrial cancer were suspected. However, the diagnosis of the complete septate uterus was confirmed only after she was operated for EC.

According to the sparse literature, the simultaneous presence of primary cancers in the endometrium and the ovary is not well documented. Approximately 5% of all patients with EC appear to have ovarian cancer synchronously, and 10% the other way around [14]. To the best of our knowledge, there are limited data to support the relationship between EC and ovarian cystadenoma.

All of these four cases of EC arising on septate uterus underwent complete staging surgery, and this present report had advanced Tanced stage III disease, and two cases had stage I disease according to the revised FIGO staging system [15]. They all involved endometrial endometrioid adenocarcinoma arising in either one or both cavities of a septate uterus. Generally, adjuvant treatment is based on the stage, grade, type of tumor, and the physical condition of the patients. However, there is no standard consensus on its therapeutic management or follow-up due to the rarity of this condition. 

## Conclusions

Uterine anomalies are rare and their diagnosis may go undetected. The coexistence of uterine malformation and EC poses the problem of confirmation in some situations and sometimes the diagnosis is made intraoperatively. Surgeons should not be surprised when they come across this unusual condition.
